# Plastin 3 in X-Linked Osteoporosis: Imbalance of Ca^2+^-Dependent Regulation Is Equivalent to Protein Loss

**DOI:** 10.3389/fcell.2020.635783

**Published:** 2021-01-21

**Authors:** Christopher L. Schwebach, Elena Kudryashova, Dmitri S. Kudryashov

**Affiliations:** Department of Chemistry and Biochemistry, The Ohio State University, Columbus, OH, United States

**Keywords:** plastin 3, X-linked osteoporosis, osteogenesis imperfecta, bone development, actin bundling, Ca^2+^-dependent regulation, PLS3, T-Plastin

## Abstract

Osteogenesis imperfecta is a genetic disorder disrupting bone development and remodeling. The primary causes of osteogenesis imperfecta are pathogenic variants of collagen and collagen processing genes. However, recently variants of the actin bundling protein plastin 3 have been identified as another source of osteogenesis imperfecta. Plastin 3 is a highly conserved protein involved in several important cellular structures and processes and is controlled by intracellular Ca^2+^ which potently inhibits its actin-bundling activity. The precise mechanisms by which plastin 3 causes osteogenesis imperfecta remain unclear, but recent advances have contributed to our understanding of bone development and the actin cytoskeleton. Here, we review the link between plastin 3 and osteogenesis imperfecta highlighting *in vitro* studies and emphasizing the importance of Ca^2+^ regulation in the localization and functionality of plastin 3.

## Introduction

Osteogenesis imperfecta (OI) is a genetic form of skeletal dysplasia affecting 1 in 10,000–20,000 live births and resulting in a broad array of clinical manifestations (Lim et al., 2017; Marini et al., 2017; Palomo et al., 2017). OI, also known as brittle bone disease, is characterized by low bone mass and increased bone fragility with severity ranging from asymptomatic to perinatal lethality (Robinson and Rauch, 2019; Rossi et al., 2019). The primary cause of most OI forms is an impaired extracellular matrix resulting from defects in either quantity/quality or assembly of collagen type I (Nijhuis et al., 2019). Approximately 85–90% of OI cases are a result of pathogenic variants of either the *COL1A1* or *COL1A2* genes (Lindahl et al., 2015). The remaining OI cases stem from variants of 18 other identified genes, the majority of which are involved in collagen type I processing (Tauer et al., 2019). Interestingly, pathogenic variants of three genes (CRTAP, P3H1, and PPIB) coding for the components of the type I collagen 3-hydroxylation complex are associated with an altered expression of lamin A/C and cofilin 1, suggesting that nucleoskeletal and cytoskeletal abnormalities may be involved in the pathogenesis of the disease (Gagliardi et al., 2017). A more direct link to the cytoskeleton is observed in X-linked OI caused by pathogenic variants in the *PLS3* gene located on the X chromosome and coding for the cytoskeletal actin-bundling protein plastin 3 (PLS3; *aka* T-plastin). To date, *PLS3* is the only OI-linked gene not known to affect collagen processing.

## PLS3 Structure and Function

**Figure 1:**
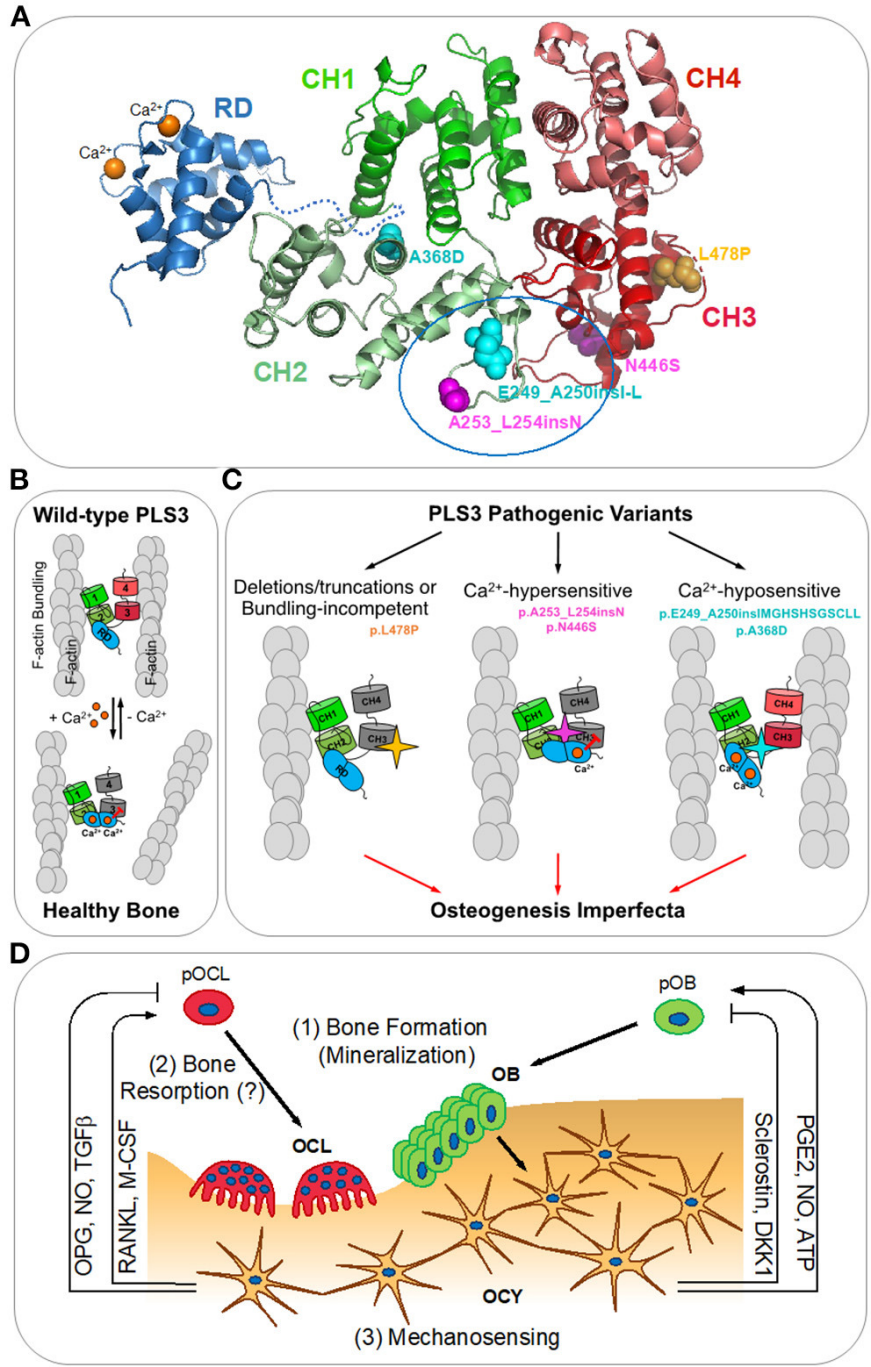
PLS3 in osteogenesis imperfecta. **(A)** A homology model of human PLS3 built using Phyre2.0 (Kelley et al., 2015) is colored according to the domain organization. Calponin-homology domains 1-2 (CH1 and CH2) and 3-4 (CH3 and CH4) form actin-binding domains 1 and 2 (ABD1 and ABD2), respectively. The N-terminal regulatory domain (RD; PDB:5JOJ; Ishida et al., 2017) is comprised of two Ca^2+^-binding EF-hand domains and positioned arbitrarily as its precise orientation relative to the actin-binding core is not known. OI-linked mutations resulting in the full-length protein are indicated as spheres: Ca^2+^-hypersensitive mutations are in magenta, Ca^2+^-hyposensitive mutations are in cyan, the bundling-disabling mutation is in yellow. A tentative RD docking site outlined by a blue oval at the ABD1-ABD2 interface has been determined by the location of the OI mutations disrupting Ca^2+^-sensitivity and reduced accessibility for labeling in the presence of the RD (Schwebach et al., 2020). **(B)** Actin bundle formation by the wild-type PLS3 is regulated by calcium. At low Ca^2+^ concentrations (nanomolar range), PLS3 forms actin bundles by binding simultaneously to two actin filaments (F-actin) via ABD1 and ABD2; saturation of the EF-hands in the RD with Ca^2+^ inhibits the ability of ABD2 to bind to F-actin and disassembles the bundle (Schwebach et al., 2017). **(C)** Finely tuned Ca^2+^ regulation of F-actin bundling by PLS3 is essential for proper bone formation (Schwebach et al., 2020). OI-causative pathogenic variants of PLS3 disrupting its normal functioning can be classified in three groups: (1) F-actin bundling-incompetent (deletions/truncations of PLS3 or p.L478P directly disrupting the actin-binding site of ABD2); (2) inhibiting ABD2 by sub-physiological Ca^2+^ concentrations (p.N446S and p.A253_L254insN); (3) disabling the ABD2 inhibition by physiological Ca^2+^ concentrations (p.A368D and p.E249_A250insIMGHSHSGSCLL). **(D)** Bone remodeling (reviewed in Niedzwiedzki and Filipowska, 2015): osteocytes (OCY) orchestrate bone lysis by osteoclasts (OCL) and bone formation by osteoblasts (OBL) via coordinated secretion of RANKL (receptor-activator of NFκβ ligand), M-CSF (macrophage colony stimulating factor), OPG (osteoprotegerin), NO (nitric oxide), TGFβ (transforming growth factor beta), PGE2 (prostaglandin E2), ATP, sclerostin, and DKK1 (Dickkopf-1). pOCL and pOBL – osteoclast and osteoblast precursors, respectively. PLS3 has been proposed to be involved in: (1) insufficient mineralization by OBL; (2) increased bone resorption by OCL; (3) dysregulation of mechanosensing by OCY leading to misbalance between bone resorption and formation.

Plastins (*aka* fimbrins) are a highly conserved family of actin-bundling proteins, which are expressed in a tissue-specific manner (Lin et al., 1993; Shinomiya, 2012). Of the three plastin isoforms expressed in vertebrates, PLS3 is the most ubiquitous isoform expressed in all solid tissues (Lin et al., 1988, 1993, 1999), while PLS1 (*aka* I-plastin) and PLS2 (*aka* LCP1, LPL, or L-plastin) are primarily found in intestinal epithelium and hematopoietic tissues, respectively. Plastins contain two actin-binding domains (ABD1 and ABD2), each comprising two calponin-homology (CH) domains (Figure 1A). Binding of both ABDs to actin filaments (F-actin) brings the filaments together into a tight bundle (Figure 1B). F-actin bundling is critical to cellular structures (such as stress fibers, filopodia, focal adhesions, and microvilli) and processes (such as cell migration, cytokinesis, endo-, and exocytosis) and appears to be the primary function of plastins (Skau et al., 2011; Schwebach et al., 2020). F-actin bundling by plastins is tightly regulated by Ca^2+^ binding to plastins' N-terminal regulatory domain (RD), which contains two Ca^2+^-binding EF-hand motifs (Figure 1A). Upon binding of Ca^2+^, the RD potently inhibits the bundling activity of plastins by disabling ABD2 (Figure 1B) (Schwebach et al., 2017, 2020).

PLS3 contributes to a variety of cellular activities and appears to be particularly important for shaping the actin cytoskeleton near membranes. Thus, PLS3 is found associated with the lamellipodium (Garbett et al., 2020; Schwebach et al., 2020) and focal adhesions in fibroblasts and osteoblasts (Schwebach et al., 2020). PLS3 has recently been shown to contribute to cell migration by strengthening and stabilizing membrane protrusions (Garbett et al., 2020). S-nitrosylation of PLS3 in endothelial cells in response to angiotensin II has been implicated in weakening adherence junctions but promoting migration and tube formation (Pan et al., 2020). Similarly, PLS2 is well-recognized to contribute to the migratory capabilities of immune and cancerous cells (Schaffner-Reckinger and Machado, 2020).

Both PLS3 and PLS2 interact with activated Rab5 and their expression is accompanied by increased fluid-phase endocytosis (Hagiwara et al., 2011). Plastin's ortholog in yeast (fimbrin) is localized almost exclusively to endocytic actin patches and is essential for endocytosis (Skau et al., 2011). Improved endocytosis has also been proposed to account for the PLS3's role as a protective modifier of spinal muscular atrophy (Ackermann et al., 2013; Lyon et al., 2014; Kaifer et al., 2017; Alrafiah et al., 2018). PLS3 also mediates membrane trafficking in hypoxia (Wottawa et al., 2017) and in the *ectoplasmic specialization*- testis-specific hybrid cell-cell junctions between Sertoli cells and spermatids. Localization of PLS3 with these contacts correlates with a highly dynamic reorganization of the membrane-associated actin cytoskeleton in spermatogenesis (Li et al., 2016).

A role of plastins in cytokinesis has also been revealed but appears to be less universal. In *Caenorhabditis elegans* zygotes, plastin increases cortical contractility and helps to stabilize myosin at the equatorial cortex (Ding et al., 2017; Leite et al., 2020), whereas the role of the fission yeast ortholog fimbrin in cytokinesis is controversial (Laporte et al., 2012; Christensen et al., 2019). Interestingly, plastin and myosin cooperate in cytokinesis (Leite et al., 2020) and epidermal morphogenesis/basement membrane assembly (Dor-On et al., 2017), while demonstrating mutually exclusive subcellular localization (Garbett et al., 2020), which can result from direct competition between myosin and plastin for binding sites on actin (Behrmann et al., 2012; Schwebach et al., 2020). Additional explanation for this antagonism is competition with tropomyosin, which has been demonstrated for yeast fimbrin (Christensen et al., 2019), but not yet for mammalian plastin and tropomyosin isoforms.

Surprisingly, PLS3 expression has also been shown to correlate with robust DNA repair (Hisano et al., 1996; Higuchi et al., 1998; Sasaki et al., 2002; Ikeda et al., 2005). Accordingly, PLS3 has been found to be an important marker in predicting the effectiveness of chemotherapeutics in the treatment of cancers (Hisano et al., 1996; Higuchi et al., 1998; Xin et al., 2020).

Despite significant advancements over the years, a comprehensive understanding of the PLS3 structure and biomechanics remains elusive. Of particular interest is obtaining a complete high-resolution structure of plastins including both the RD and actin-binding core, as this discovery would aid in deciphering a detailed molecular mechanism of PLS3 regulation by Ca^2+^. Furthering our understanding of the molecular and structural aspects of PLS3 bundling and regulation will be critical to uncover the role(s) of PLS3 in cells and tissues and shed light on how pathogenic variants of *PLS3* lead to OI. It remains intriguing and obscure how mutations in the ubiquitously expressed cytoskeletal protein PLS3 result in pathogenic phenotype exclusively in bone tissue. A possible explanation is partial functional redundancy with other plastin isoforms (or other actin-bundling proteins). In this regard, it will be important to analyze the expression patterns of the less ubiquitous PLS1 and PLS2 isoforms in different tissues in response to the impact from the pathogenic PLS3 variants.

## PLS3 Pathogenic Variants Identified in X-Linked Osteogenesis Imperfecta and Their Clinical Manifestations

To date, 27 OI-associated pathogenic variants (including coding mutations and gene deletions/truncations) have been identified in the *PLS3* gene located on the X chromosome (Table 1). The majority of these are expected to result in the absence of functional protein: four full or partial *PLS3* gene deletions (Kämpe et al., 2017a; Kannu et al., 2017; Lv et al., 2017), seven frame-shift mutations resulting in truncated mRNA constructs (Fahiminiya et al., 2014; Nishi et al., 2016; Kämpe et al., 2017a,b; Lv et al., 2017), six nonsense mutations (Fahiminiya et al., 2014; Kämpe et al., 2017b; Balasubramanian et al., 2018; Chen et al., 2018; Wang et al., 2020), and one aberrant splicing variant (Cao et al., 2019). All but two of the resulting truncated mRNA molecules appear to be the canonical substrates for degradation by nonsense-mediated mRNA decay (NMD) (Miller and Pearce, 2014; Kishor et al., 2019). Indeed, in several tested cases, the pathogenic variants of PLS3 have not been detected at the protein level in tissues from OI patients (Van Dijk et al., 2013; Wang et al., 2020). The two variants, which do not meet the criteria for degradation by the NMD pathway may generate truncated proteins, which, however, are highly unstable upon expression in *Escherichia coli* and are unlikely to be functional when expressed *in vivo* (Schwebach et al., 2020).

**Table 1:** List of identified OI-linked PLS3 variants.

**cDNA^**a**^**	**Protein**	**Mutation type**	**Expression**	**References**
c.73-24T>A	p.D25AfsX17	Frameshift (splice variant)	Not investigated	Laine et al., 2015
c.235delT	p.Y79IfsX6	Frameshift	No expression	Van Dijk et al., 2013
c.244C>T	p.Q82X	Non-sense	No expression	Wang et al., 2020
c.321T>A	p.G107=	Synonymous	Normal expression	Van Dijk et al., 2013
c.745G>T	p.E249X	Non-sense	Not investigated	Chen et al., 2018
c.748+1G>A	p.E249_A250ins-IMGHSHSGSCLL	Insertion (splice variant)	Decreased expression^b^	Van Dijk et al., 2013
c.759_760insAAT	p.A253_L254insN	Insertion	Normal expression^b^	Van Dijk et al., 2013
c.766C>T	p.R256X	Non-sense	Not investigated	Kämpe et al., 2017b
c.892-1G>A		splice variant	Not investigated	Cao et al., 2019
c.925A>G	p.I309V	Missense	Not investigated	Kämpe et al., 2017b
c.994_994delGA	p.D332X	Non-sense	Not investigated	Fahiminiya et al., 2014
c.1103C>A	p.A368D	Missense	Not investigated^b^	Nishi et al., 2016
c.1096_1100delAACTT	p.N366SfsX5	Frameshift	Not investigated	Costantini et al., 2018
c.1106_1107insGAAA	p.F369LfsX5	Frameshift	Not investigated	Hu et al., 2020
c.1242T>C	p.P414=	Synonymous	Not investigated	Kämpe et al., 2017b
c.1294T>C	p.L432=	Synonymous	Not investigated	Kämpe et al., 2017b
c.1295T>A	p.L432X	Non-sense	Not investigated	Balasubramanian et al., 2018
c.1424A>G	p.N446S	Missense	Not investigated^b^	Kämpe et al., 2017b
c.1433T>C	p.L478P	Missense	Not investigated^b^	Fahiminiya et al., 2014
c.1471C>T	p.Q491X	Non-sense	Not investigated	Van Dijk et al., 2013
c.1647delC	p.S550AfsX9	Frameshift	Not investigated	Van Dijk et al., 2013
c.1730dupT	p.T578NfsX4	Frameshift	Not investigated^c^	Kannu et al., 2017
c.1765del	p.A589QfsX21	Frameshift	Not investigated^c^	Balasubramanian et al., 2018
		Gene deletion	None	Kannu et al., 2017
		Gene deletion	None	Kämpe et al., 2017a
		Exon 4–16 deletion	None	Kämpe et al., 2017a
		Exon 10–16 deletion	None	Lv et al., 2017

a *Numbering based on reference sequence NM_005032.7*.

b *In vitro, the mutated proteins were soluble, but had slightly reduced stability (Schwebach et al., 2020)*.

c *In vitro, the truncated proteins were largely insoluble, prone to degradation, and had significantly reduced stability (Schwebach et al., 2020)*.

Of particular interest in further understanding of PLS3's role in OI are variants that generate full-length mutated proteins. In two cases, the nucleotide insertions do not result in a frame shift and give rise to full-length PLS3 protein variants containing one (p.A253_L254insN) or twelve (p.E249_A250insIMGHSHSGSCLL) extra amino acids (Van Dijk et al., 2013). In three other cases, full-length OI-linked proteins result from missense mutations p.A368D, p.N446S, and p.L478P (Fahiminiya et al., 2014; Nishi et al., 2016; Kämpe et al., 2017b). Structural and functional consequences of these variants are discussed below.

The severity of OI clinical manifestations does not appear to directly correlate with a specific pathogenic *PLS3* variant and varies greatly from the absence of physical symptoms to severe osteoporosis and dysmorphia (Makitie et al., 2019). While all patients have classical signs of osteoporosis with thin trabeculae and scarce osteoid seams, in general, hemizygous males are more affected than heterozygous females (Makitie et al., 2019). Phenotypes vary in females from mild to severe with variations existing within families (Kämpe et al., 2017b), which may be explained by variability in inactivation of the mutated X chromosome. Low bone mineral density (BMD) is common for all *PLS3* OI variants, however its extent varies. Bone fractures are a functional consequence of decreased BMD and constitute another universal trait in PLS3-related OI. Vertebrae compression fractures, as well as long bone fractures resulting from minor trauma are common. In more severe cases, phenotypes can include facial dismorphism, clumsy gait, joint hyperlaxity, kyphosis, deafness, and blue sclerae (Fahiminiya et al., 2014; Nishi et al., 2016; Kämpe et al., 2017a,b; Lv et al., 2017; Chen et al., 2018; Costantini et al., 2018; Cao et al., 2019; Hu et al., 2020).

## Importance of PLS3 for Proper Bone Formation

Animal model studies have emphasized an importance of PLS3 in bone tissue development and support of healthy bone architecture. A *Pls3* knock down in zebrafish manifests in significant skeletal and muscular abnormalities (Van Dijk et al., 2013), while *Pls3* knock out mice show significant osteoporosis and decreased bone strength mirroring human OI symptoms (Neugebauer et al., 2018; Yorgan et al., 2019). Concordantly, PLS3 over expression in mice has resulted in increased bone strength and a thickened cortical bone (Neugebauer et al., 2018). However, the molecular mechanisms underlying the PLS3 function in bone tissue and whether PLS3 actin-bundling ability is important for bone homeostasis remain elusive.

To date, few studies have addressed the mechanisms by which *PLS3* variants cause OI. Strong hypomineralization of the bone matrix is a common finding but is not thought to be due to increased bone turnover, albeit evidence of increased bone resorption has been reported (Laine et al., 2015). Bone shows reduced mineralizing surface and adjusted mineral apposition rate, while mineralization lag time is significantly increased (Laine et al., 2015; Kämpe et al., 2017a). A link between PLS3 and mineralization has been supported by increased expression of PLS3 in osteoblastic MC3T3-E1 cells upon mineralization (Fahiminiya et al., 2014) as well as the presence of PLS3 in matrix vesicles (Thouverey et al., 2011; Kim et al., 2013) generated from the apical microvilli of osteoblasts during mineralization (Thouverey et al., 2011). The presence of PLS3 in matrix vesicles correlates with its function as a regulator of apical microvilli (Fath and Burgess, 1995; Volkmann et al., 2001; Delanote et al., 2005) and membrane trafficking (Hagiwara et al., 2011; Wottawa et al., 2017). However, it remains unknown whether PLS3 contributes to the role of matrix vesicles in bone mineralization.

## Calcium Regulation Is Key to PLS3 Activity and Drives Its Localization in Cells

A recent study has revealed the molecular consequences of OI-linked full-length PLS3 variants (p.E249_A250insIMGHSHSGSCLL, p.A253_L254insN, p.A368D, p.N446S, and p.L478P) at the protein and cellular levels (Schwebach et al., 2020). These variants segregate into three groups (Figure 1C) with distinct biochemical properties and intracellular localization. The p.L478P variant has been found to bind actin through intact ABD1 while being unable to bundle actin filaments *in vitro*. High-resolution cryo-EM reconstruction of ABD2 bound to F-actin has provided structural insights for this deficiency: the L478P mutation disrupts the structure of a conserved loop involved in F-actin binding. In cells, wild-type PLS3 has been shown to localize to actin-rich structures in lamellipodia and focal adhesions. This localization is severely affected for the bundling-deficient p.L478P, which, despite actin binding via ABD1, failed to associate with the actin cytoskeleton. This finding implies that F-actin bundling, rather than binding, is the primary function of PLS3 required for its proper intracellular localization including its association with the branched actin network at the leading edge. Therefore, similar to other loss-of-function variants (frame-shift, non-sense, and *PLS3* gene deletions), the L478P missense mutation results in non-functional (i.e., non-bundling) PLS3 as a likely cause of OI.

More intriguingly, not only is PLS3 actin-bundling activity important, but its finely tuned Ca^2+^ regulation is essential for proper bone formation (Schwebach et al., 2020). Indeed, it has been demonstrated that the remaining full-length OI-causative PLS3 variants are capable to bundle actin, but their response to Ca^2+^ is perturbed toward either hypersensitivity (p.A253_L254insN and p.N446S) or hyposensitivity (p.E249_A250insIMGHSHSGSCLL and p.A368D). The altered sensitivity to Ca^2+^ affects the localization of PLS3 within cells: variants hypersensitive to Ca^2+^ associate more strongly with the branched actin network in the lamellipodia and are largely excluded from focal adhesions, while Ca^2+^-insensitive variants are depleted from the lamellipodia and localize primarily at significantly enlarged focal adhesions and in stress fibers. Moreover, such differential localization of PLS3 is Ca^2+^-dependent, as Ca^2+^ depletion from the cell culture medium has resulted in redistribution of PLS3 from the lamellipodia to focal adhesion sites, implying that Ca^2+^ aids in cycling of PLS3 between aligned actin bundles in focal adhesions and the branched actin network at the leading edge. This suggests that fine regulation of PLS3 by Ca^2+^ is critical to bone formation as its imbalance in either direction results in OI, comparable to that resulting from the loss of PLS3 (Figure 1C).

It should be restated that to date, no complete structure of any plastin/fimbrin has been described and the location of the RD relative to the core remained unknown until recently. While the structures of the actin-binding core (Klein et al., 2004) and the regulatory domain (Ishida et al., 2017) have been solved, a molecular model of how they are associated with each other has not been produced. Analysis of four distinct OI-linked PLS3 variants resulting in opposing Ca^2+^ sensitivities has suggested that the RD is positioned at the interface between ABD1 and ABD2. Homology modeling suggests that flexible loops between CH1-CH2 (containing residues E249-A250) and CH2-CH3 reside in proximity and together likely represent the RD docking site (Figure 1A). This hypothesis has been confirmed by differential labeling of two designed cysteine residues in the absence and presence of the RD (Schwebach et al., 2020).

Molecular dynamics simulations of one PLS3 variant, p.E249_A250insIMGHSHSGSCLL, has predicted the conformational changes in a loop between CH1 and CH2 in ABD1 (Chen et al., 2018). Chen and coauthors suggest that, despite the addition of 12 amino acids, the mutant showed less conformational change than the wild-type protein and a more compact structure via a tighter association of the loop with CH3 of ABD2 (Chen et al., 2018). The simulation, however, used a homologous model of the protein and, therefore, must be revised after the high-resolution structure becomes available.

## Potential Mechanisms by Which *PLS3* Pathogenic Variants Perturb Osteogenesis

The mechanisms by which PLS3 affects osteogenesis/bone remodeling remain largely unknown. Three main hypotheses of PLS3-related osteogenesis imperfecta mechanisms have emerged (Figure 1D): (1) insufficient mineralization by osteoblasts (Fahiminiya et al., 2014), (2) increased bone resorption by osteoclasts (Neugebauer et al., 2018), and (3) dysregulation of osteocyte mechanosensing leading to misbalance between bone resorption and formation (Van Dijk et al., 2013).

(1) The effect of PLS3 on bone mineralization by osteoblasts has been inferred in multiple studies focusing on the level of bone mineralization in patients. Fahiminiya and colleagues found that *in vitro* differentiation of cultured mouse cranial MC3T3-E1 osteoblasts correlated with the increased expression of PLS3 (Fahiminiya et al., 2014), indirectly implying that PLS3 may be involved in bone mineralization. *PLS3* deletions have been shown to cause significant hypomineralization of the bone matrix (Kämpe et al., 2017a). This is in contrast to OI caused by *COL1A1* or *COL1A2* variants, which result in hypermineralized bone matrix (Roschger et al., 2008). Regulation of intracellular vesicles in late osteoblasts transitioning to early osteocytes is thought to drive bone mineralization (Hearn and Russell, 1981; Barragan-Adjemian et al., 2006). PLS3 is recognized as a regulator of vesicle trafficking (Hagiwara et al., 2011; Wottawa et al., 2017) and is upregulated in matrix vesicles and microvilli of osteoblasts upon mineralization (Thouverey et al., 2011; Kim et al., 2013) further suggesting a link between *PLS3* variants and altered bone matrix mineralization.

(2) Contribution of PLS3 to bone resorption would be surprising given that osteoclasts as all cells of hematopoietic origin express PLS2 as the primary plastin isoform (Ma et al., 2010; Si et al., 2018; Li et al., 2020). Thus far, endogenous expression of PLS3 has not been clearly demonstrated in osteoclasts *in vivo*. Accordingly, PLS3 has been shown to have no effect on osteoclast activity including bone resorption (Fahiminiya et al., 2014; Kämpe et al., 2017a). Yet, more recent work has proposed a role for PLS3 in osteoclast activity through the regulation of podosomes by NFκB signaling (Neugebauer et al., 2018). Since these findings are based on either overexpression or knockout of *PLS3* in mouse models, evaluation of the effects of endogenously expressed pathogenic variants of *PLS3* would be required to verify the validity of this hypothesis.

(3) Involvement of PLS3 in mechanosensing by osteocytes has also been hypothesized (Van Dijk et al., 2013; Laine et al., 2015). Recent studies demonstrating the importance of Ca^2+^ regulation in the PLS3 actin-bundling activity (Schwebach et al., 2017, 2020) have added further credence to this hypothesis. Embedded in bone matrix, osteocytes orchestrate bone remodeling by coordinated secretion of factors regulating both bone lysis by osteoclasts and bone construction by osteoblasts (reviewed in Niedzwiedzki and Filipowska, 2015) (Figure 1D). Therefore, being the major plastin isoform in osteocytes, PLS3 could contribute to both osteogenesis and osteolysis. Intriguingly, PLS3 is enriched in dendrites, and specifically at their branching points (Kamioka et al., 2004), which are recognized as the structures by which osteocytes sense their environment (Kamioka et al., 2004; Galli et al., 2010). Furthermore, the activity of integrin/adhesion-dependent voltage-gated calcium channels is a strong candidate for the driving force of bone formation and remodeling (O'Neill and Galasko, 2000; Li et al., 2002; Cao et al., 2019; Sun et al., 2019). The findings that Ca^2+^ is involved in the redistribution of PLS3 from focal adhesions to the leading edge (Schwebach et al., 2020) represents a strong link between the activities of PLS3 and the machinery thought to drive bone mechanosensing and reorganization. Future work in osteocytes is required to determine the specific role of PLS3 and its regulation by Ca^2+^ in these activities.

## Conclusion

PLS3-mediated osteogenesis imperfecta is an exciting new field of study with implications in the clinic as well as the fields of bone development and actin biochemistry. Uncovering the mechanisms by which PLS3 causes OI has already contributed to our understanding of PLS3's roles and regulation in cells as well as guiding ongoing structural work. Future discoveries testing the hypotheses described above should reveal not only the mechanisms of X-linked osteoporosis but more broadly answer lingering questions about bone mineralization and mechanosensing. Integration of future PLS3 biochemistry and bone development studies could lead to targeted therapeutics for X-linked OI.

## Author Contributions

CLS designed and drafted the manuscript and prepared the table. EK and DSK edited the manuscript and prepared the figures. All authors revised the manuscript and approved the submitted version.

## Conflict of Interest

The authors declare that the research was conducted in the absence of any commercial or financial relationships that could be construed as a potential conflict of interest.
